# Redox status of cysteines does not alter functional properties of human dUTPase but the Y54C mutation involved in monogenic diabetes decreases protein stability

**DOI:** 10.1038/s41598-021-98790-3

**Published:** 2021-09-28

**Authors:** Judit Eszter Szabó, Kinga Nyíri, Dániel Andrási, Judit Matejka, Olivér Ozohanics, Beáta Vértessy

**Affiliations:** 1 Institute of Enzymology, RCNS, Eötvös Loránd Research Network, Budapest, Hungary; 2 Department of Applied Biotechnology and Food Sciences, Budapest University of Technology and Economics, Budapest, Hungary; 3 Department of Biochemistry, Institute of Biochemistry and Molecular Biology, Semmelweis University, Budapest, Hungary

**Keywords:** Biochemistry, Enzyme mechanisms, Proteins, Structural biology

## Abstract

Recently it was proposed that the redox status of cysteines acts as a redox switch to regulate both the oligomeric status and the activity of human dUTPase. In a separate report, a human dUTPase point mutation, resulting in a tyrosine to cysteine substitution (Y54C) was identified as the monogenic cause of a rare syndrome associated with diabetes and bone marrow failure. These issues prompt a critical investigation about the potential regulatory role of cysteines in the enzyme. Here we show on the one hand that independently of the redox status of wild-type cysteines, human dUTPase retains its characteristic trimeric assembly and its catalytic activity. On the other hand, the Y54C mutation did not compromise the substrate binding and the catalytic properties of the enzyme at room temperature. The thermal stability of the mutant protein was found to be decreased, which resulted in the loss of 67% of its activity after 90 min incubation at the physiological temperature in contrast to the wild-type enzyme. In addition, the presence or absence of reducing agents had no effect on hDUT^Y54C^ activity and stability, although it was confirmed that the introduced cysteine contains a solvent accessible thiol group.

## Introduction

Preservation of genome integrity is essential for viability and is accomplished by efficient interlinked regulatory pathways of DNA damage recognition / repair and nucleotide biosynthesis. In eukaryotes, these processes are generally compartmentalized in the nucleus and in the mitochondrion. These two organelles represent altered redox milieu, as shown by several independent experimental approaches (e.g. refs^1–3^, cf also for a review^4^). It is also of importance to provide functionality of organelle-specific genomic repair and nucleotide biosynthesis in line with the actual redox status (cf e.g. refs^5–9^, cf also for a review^10^).

**Figure 1:**
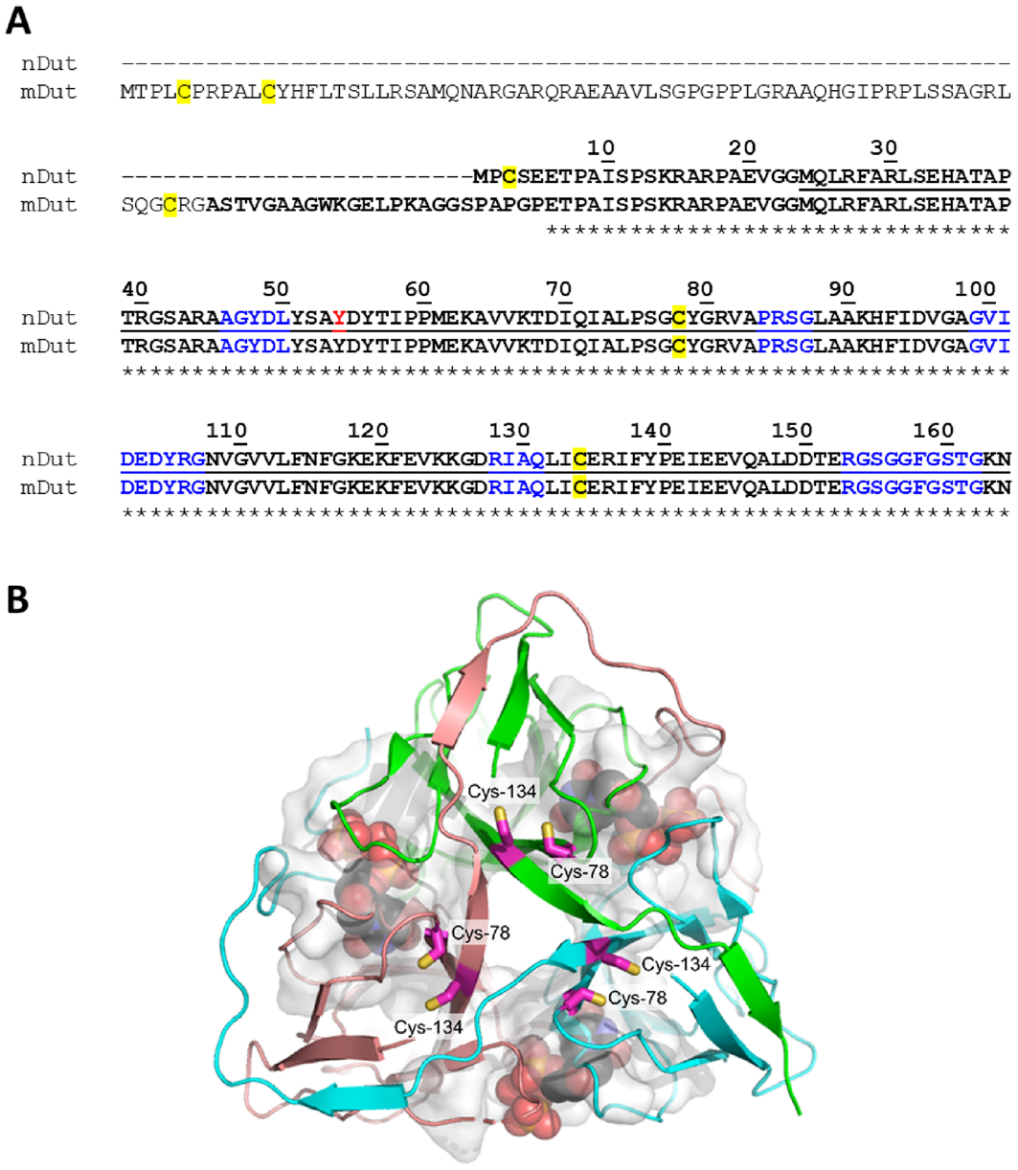
(**A**) Sequences of the nuclear (nDut) and mitochondrial (mDut) isoforms of the human dUTPase (Uniprot ID: P33316-2 and -3). Numbering of nuclear isoform of the human dUTPase residues is shown above the sequences. Stars denote the identical residues. In case of mDut the functional protein sequence remaining after the removal of mitochondrial localization sequence is shown in bold. Residues included in the crystal structure shown on Panel A are underlined. Cysteines are with yellow outline, position of Y54C mutation is coloured by red. Residues of the five conserved motif are coloured blue. (**B**) Crystal structure of the human dUTPase (PDB ID: 2HQU). Protein is shown as cartoon, chains are coloured cyan, salmon and green. Substrate analogue 2ʹ-deoxyuridine 5ʹ-alpha,beta-imido-triphosphate (dUPNPP) is shown as spheres with atomic coloring (carbon: black, oxygen: red, nitrogen:blue, phosphorus:orange). Residues within 5 Å distance from the substrate analogue are shown as white partially transparent surface. Cysteine side chains are shown as sticks with atomic coloring (carbon: magenta, sulfur: yellow), labels of cysteines are shown. Both Cys-78 and Cys-134 residues are in the globular core of the protein more than 5 Å distance from the active site of the protein.

The dUTPase enzymes play a dual role in sanitizing the nucleotide pool and in providing precursor for thymidylate biosynthesis^11,12^. It has been established that in humans and in several other eukaryotes, two dUTPase isoforms are present, one nuclear and one mitochondrial and their expression is under control of alternative promoters^13,14^. The human dUTPase isoforms differ only in their respective intracellular trafficking signals: a well characterized nuclear localization signal^13,15^ for the nuclear isoform and a mitochondrial leader sequence for the mitochondrial isoform^13^ (Fig. 1A). With regard to the different redox status of the nucleus and the mitochondrion, it is important to discuss the location of cysteine residues in the structure. There are two cysteine residues (Cys78 and Cys134) within the conserved major part of the human dUTPase isoforms, which are buried within the well folded structure, more than 5 Å distance from the active site. There is one additional cysteine on the flexible N-terminus of the nuclear isoform (Cys3), which could not be included in the 3D structure based on X-ray crystallographic data as the electron density map is poorly defined around this mobile section of the protein^16^ (Fig. 1B). The mature mitochondrial isoform that is produced after the mitochondrial import by cleaving the organelle-specific leader segment, does not contain this latter, potentially solvent exposed cysteine.

dUTPases belong to two enzyme families, the all-alpha and the all-beta dUTPase family^11,17^. Most dUTPases, including mammalian dUTPases are all-beta dUTPases^18^. All-beta dUTPases are homotrimers, constituted by intrinsically folded beta-pleated subunits^13,19–37^. The three active sites are found within the clefts formed by subunit interfaces. In most cases, the C-terminal part of a given subunit folds back to the remote active site in such a manner that each active site involves conserved residues from all the three subunits (Fig. 1B). This fold is highly specific and is strictly conserved in the trimeric dUTPase family. The well-folded core is frequently complemented by lineage-specific N-terminal and C-terminal flexible extensions^38,39^, or by intrastrand extensions that fold out from the core^24,29,32,35,40^. In eukaryotes the flexible N-termini usually encode organelle specific signal sequences for intracellular trafficking^11,13,38^.

It has been proposed that the N-terminal cysteine of the nuclear isoform leads to formation of a covalently linked dimer through a disulfide bridge between Cys3 residues with regulatory consequences^41^. Rotoli et al*.* also claimed that this disulfide bridge is essential for activity based on experiments indicating inactivation of the enzyme by the Cys3Ala mutation or by the addition of excess reducing agents^41^. The proposal of an enzymatically active covalently linked dimeric state of human dUTPase is highly intriguing. However, it cannot be reconciled in the current structure–function knowledge on this enzyme, which clearly indicated an indispensable condition for the homotrimeric assembly to form functional active sites based on all available 3D crystal structure data and solution studies^13,19–37,42,43^. Although an artificial covalent dimer construct of *Drosophila virilis* dUTPase could have been created, it showed decreased enzymatic activity as compared to the respective trimeric enzyme^21^.

These results of Rotoli et al*.* are also in contradiction with data reported for full enzymatic activity preserved in an N-terminally truncated human dUTPase construct (M24-N164) lacking the flexible N-terminus and the Cys3 residue located within. The k_cat_ for this enzyme construct was reported as 8.2 ± 0.6 s^−1^ (37 °C)^19^ to be compared with k_cat_ = 6.8 ± 2.0 s^−1^ (20 °C) for the full-length and His-tagged protein^44^. Also, limited trypsinolysis leading to cleavage of the flexible N-terminus of human dUTPase did not lead to any loss of enzymatic activity^16^.

dUTPase is an enzyme essential for viability and its regulation is of key importance, therefore in the present study we set out to shed light on this contradiction. The essential character of dUTPase has been well established in most free-living species^40,45–50^ investigated so far, with some potential exceptions to this general rule in some prokaryotes^51^. Potentially in line with the importance of dUTPase in cell viability, no human dUTPase knock-outs could have been obtained to date. However, silencing studies reinforced the essentiality of human dUTPase and its key role in regulating thymidylate metabolism^31,52–55^. It has also been shown that dUTPase overexpression in cancers decrease the effectivity of thymidylate-synthase targeted chemotherapy (e. g. 5-fluorouracil, methotrexate)^54–57^ and is involved in development of resistance against the treatment^58,59^. Interestingly, a human dUTPase mutation (Tyr54Cys) located outside the conserved region was suggested to be responsible for monogenic syndrome associated with diabetes type 2 and bone marrow failure^53^. This report was the first and to date the only case where a dUTPase mutation has been associated with human disease. It was suggested that the mutation affects enzymatic activity, however, no kinetic data was reported. It was therefore of immediate interest to characterize the structural and functional consequences of this mutation.

## Results

### Redox status of cysteines does not alter functional properties of human dUTPase

**Figure 2:**
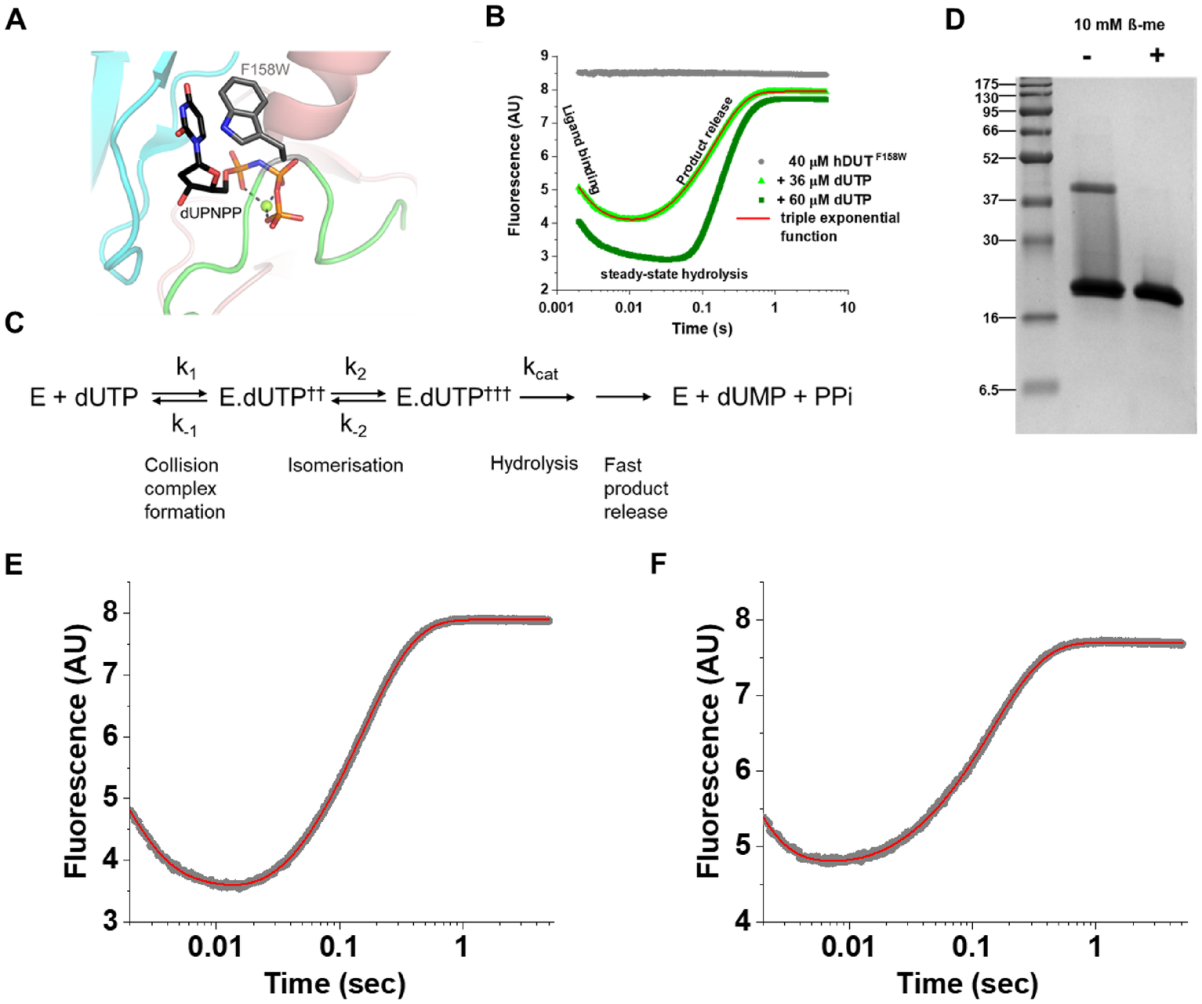
(**A**) Model structure of the F158W mutant human dUTPase. The model was created with PyMOL Mutagenesis tool from the crystal structure of the human dUTPase (PDB ID: 2HQU). Protein is shown as cartoon, chains are coloured cyan, salmon and green. Substrate analogue dUPNPP is shown as sticks with atomic coloring (carbon: black, oxygen: red, nitrogen: blue, phosphorus: orange). The sidechain of the introduced tryptophan sensor (F158W) is shown as sticks with atomic coloring (carbon: grey, nitrogen: blue). Magnesium ion is shown as green sphere, black dashed lines represent the polar contacts of the magnesium ion with the phosphate chain of the substrate analogue dUPNPP. The built in F158W tryptophan sensor can detect the presence or the absence of the substrate or the substrate analogue, thus the reaction can be followed by tryptophan fluorescence as shown on Panel B. (**B**) Representative transient kinetic reaction curves of hDUT^F158^^W^ followed by tryptophan fluorescence. The bright green reaction curve was recorded under single turnover (substrate concentration < active site concentration), while the dark green reaction curve under multiturnover conditions. The fluorescence decreases upon substrate binding and increases upon substrate/product release. The product release is rate limited by the hydrolysis event (see Panel C also). Under multiturnover conditions, a longer low fluorescent phase equivalent to steady-state hydrolysis can be observed. Single turnover curves can, while the multiturnover reaction curves can not be fitted with triple exponential function. (**C**) Schematic representation of dUTPase kinetic mechanism. The “†” marks represent fluorescent quenching. The rate constants of the steps which can be detected by tryptophan fluorescence in stopped flow under single turnover conditions are indicated on the reaction scheme. However, under single turnover conditions the rate constants of the collision complex formation (k_1_, k_−1_) and the isomerization (k_2_, k_−2_) can not be directly determined. Under these conditions only “observed binding rate constants” can be detected (k_1obs_, k_2obs_) that are originated from the net result of the forward and backward reaction of the given step, and are characteristic for the used enzyme and substrate concentration. (**D**) SDS-PAGE gel of the hDUT^F158W^ protein in the absence and presence of 10 mM β-ME. (Full-length gel image is included in a Supplementary Information.) (**E–F**) Representative single turnover fluorescence reaction curves of hDUT^F158W^ in the presence (**E**) and in the absence (**F**) of 10 mM β-ME. The reaction curves were fitted with triple exponential function (Eq. 1) which yielded the following parameters A_1_ = 3.95 ± 0.11, A_2_ = 1.15 ± 0.01, A_3_ = − 5.02 ± 0.004, k_1_ = 730.2 ± 15.3, k_2_ = 96.6 ± 1.5, k_3_ = 6.58 ± 0.01, y_0_ = 7.89 ± 0.0009 for Panel E and A_1_ = 4.12 ± 0.36, A_2_ = 0.39 ± 0.04, A_3_ = − 3.14 ± 0.003, k_1_ = 1024.3 ± 55.0, k_2_ = 193.9 ± 14.4, k_3_ = 7.0 ± 0.01, y_0_ = 7.70 ± 0.0009 for Panel F (the errors represent the error of the fitted parameters). Several (3–5) single turnover reaction curves were recorded, and the average and standard deviation of the resulted rate constants are shown in Table 1.

First, we set out to explore whether the function of human dUTPase is changed depending on the reducing or non-reducing experimental conditions. For these investigations, we chose the hDUT^F158^^W^ quasi-wild-type human dUTPase variant, which has the same sequence as the nuclear isoform (Fig. 1A, nDut), but a tryptophan sensor replaces Phe-158 at its active site (Fig. 2A). The effect of this tryptophan has been characterized in the literature^16,44,60^. The F/W substitution in human dUTPase was found not to affect the activity and substrate binding properties of the enzyme, hence this construct is considered to be a quasi-wild-type human dUTPase. Besides, the tryptophan substitution allowed the exploration of the kinetic mechanism of this enzyme, since the aromatic residue serves as a sensitive sensor to follow substrate/product binding and release and thus the enzymatic cycle of human dUTPase, even on the millisecond scale (Fig. 2B,C).

We purified this protein in the absence of any reducing agent. The SDS-PAGE gel of this enzyme preparation (Fig. 2D) indicated that in addition to the expected protein band at the ~ 19 kDa position (corresponding to a protein monomer), an extra band was also observed at around 38 kDa. This position matches with the molecular mass of a human dUTPase dimer.

As it was suggested by Rotoli et al*.* human dUTPase dimers may be formed by cysteine disulfide bonds in the absence of reducing agent. To see whether the second band is due to covalent intersubunit cysteine bridges, we added 10 mM 2-mercaptoethanol (β-ME) to the solution. Elimination of the band at 38 kDa position by addition of reducing agent (Fig. 2D) suggested that this band indeed resulted from covalent intersubunit link formation through cysteines. Thus, we observed that intersubunit disulfide bond between two human dUTPase subunits can be detected in the absence of reducing agent, although this observation was reached under denaturing conditions, i.e. by SDS-PAGE. It has to be noted that under these conditions the protein chain is unfolded, hence the covalent dimer may not be relevant for the native state (i.e. it may be an artefact). In our former SAXS experiments the nuclear isoform of the human dUTPase could only be detected as trimers in solution^42,43^. This result does not exclude the possibility of an intersubunit disulfide bond through Cys3 residues between two subunits within one trimer. Such an intersubunit disulfide bond would result in a dimer band on a non-reducing SDS-PAGE gel.

To gain relevant data about oligomerization of hDUT under native conditions we also investigated the oligomeric state of hDUT with native mass spectrometry (MS) and chemical crosslinking experiments (Supplementary Fig. 1). Trimers were the most abundant form of hDUT^F158^^W^ (61%) in the mass spectra; monomers were also detected in considerable amount (36%), while only few dimers were present (3%) (Supplementary Fig. 1A). The SDS-PAGE analysis of purified hDUT^F158W^ samples crosslinked with disuccinimidyl suberate, an agent of 11 Å-spacer length, indicated crosslinked trimers, dimers and monomers as well (Supplementary Fig. 1B). In contrast to our present crosslinking results, when formaldehyde crosslinking experiment was performed on human cells, no trimers from the human dUTPase nuclear isoform were observed^41^. We propose that this contradiction is due to the small size of formaldehyde (zero-length crosslinker), which limits its ability to crosslink larger assemblies and the low amount of dUTPase protein within the cell. Based on our results acquired with three independent experimental methods (previous SAXS results^43^, together with the present native mass spectrometry and crosslinking experiments), we suggest that the trimeric form of the nuclear isoform of human dUTPase is abundant in solution.

To test whether the presence of the reducing agent influences the activity of human dUTPase we performed transient kinetic experiments. If the active site concentration does not exceed the substrate concentration, then only one substrate molecule will be processed by one active site (single turnover condition). If the protein and substrate concentration is high enough to prevent the rate limitation of complex formation then under single turnover conditions the intrinsic catalytic rate constant of human dUTPase can be determined by triple exponential fitting (Eq. 1)^44^.

According to our results (Fig. 2E,F; Table 1) the triple exponential function could be fitted to the reaction curve where the enzyme:substrate ratio is 1:1, indicating that (i) the enzymatic mechanism of hDUT^F158^^W^ remains the same under both reducing and non-reducing conditions, and (ii) 100% of the protein is active under both conditions. We presume that the trimer state is prevalent, as the applied assay buffer is highly similar to the one previously used in non-reducing SAXS experiments^43^, where only the trimeric form of the enzyme could be detected. Moreover, based on the current structure function knowledge the homotrimeric assembly is necessary for active site formation (cf. for example refs^19,44^). The 100% activity argues for negligible amount of inactive monomers or dimers of hDUT^F158W^ under both conditions.

**Table 1:** Enzymatic parameters of hDUT^F158^^W^ and the hDUT^F158W,Y54C^ in the presence and absence of reducing agent (Kinetic data represent the average and standard deviation of 3–5 measurements, T_M_ data represent the average and standard deviation of 3 parallels).

		hDUT^F158^^W^		hDUT^F158^^W,Y54C^	
		Reducing agent*			
		+	−	+	**−**
Stopped-flow single turnover	k_1obs_ (s^−1^)	766 ± 72	847 ± 161	892 ± 212	784 ± 162
	k_2obs_ (s^−1^)	111 ± 25	119 ± 125	123 ± 54	72 ± 37
	k_cat_ (s^−1^)	**6.7 ± 0.2**	**7.2 ± 0.3**	**7.0 ± 0.3**	**7.0 ± 0.2**
T_M_	°C	58.5 ± 0.5	59.0 ± 0.5	52.5 ± 0.5	52.5 ± 0.5

*10 mM β-ME.

Regarding rate constants involved in formation of enzyme:substrate complex, under single turnover conditions two “observed binding rate constants” can be determined (k_1obs_, k_2obs_). These observed rate constants (k_1obs_ and k_2obs_) are related to the intrinsic rate constants (Fig. 2B,E,F)^44^, however these are concentration dependent. Therefore, we have used the same enzyme and substrate concentrations for reducing and non-reducing conditions to allow correct comparisons. We could not observe any remarkable difference, as presented in Table 1.

We conclude, that even if a covalent link between two subunits of a homotrimer is present in the solution through cysteines under non-reducing conditions, the measured kinetic properties do not differ. Thus, it does not seem likely that the cysteines of human dUTPase would work as a redox switch.

### Y54C mutation involved in monogenic diabetes decreases protein stability

**Figure 3:**
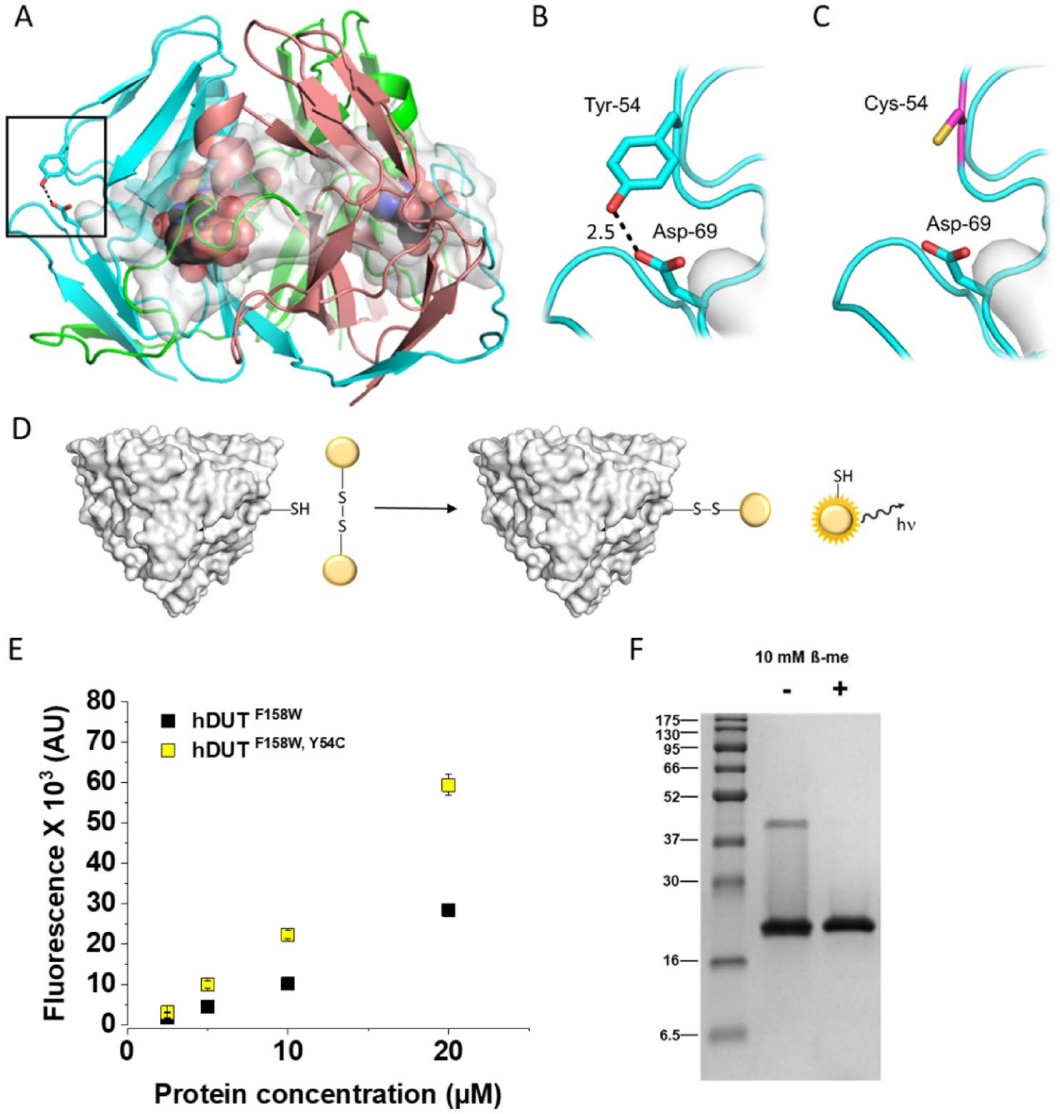
(**A**) Crystal structure of the human dUTPase (PDB ID: 2HQU). Protein is shown as cartoon, chains are coloured cyan, salmon and green. Substrate analogue dUPNPP is shown as spheres with atomic coloring (carbon: black, oxygen: red, nitrogen: blue, phosphorus: orange). Residues within 5 Å distance from the substrate analogue are shown as white partially transparent surface. In case of one of the dUTPase protomers side chains of Tyr-54 and its interaction partner Asp-69 are shown as sticks with atomic coloring (carbon: cyan, oxygen: red). Tyr-54 and Asp-69 residues are more than 5 Å distance from the active site of the protein. Black frame shows the position of close-ups on Panels B and C. (**B**) Close up of Tyr-54 in one of the dUTPase protomers. Coloring and representation is as on Panel A. Dashed black lines illustrate the hydrogen bond formed between Tyr-54 and Asp-69 (distance of the two residues in Å is indicated on the figure). (**C**) Close up of the introduced Cys-54 mutation to the protein with PyMOL in most abundant conformation (> 80%) of the residue (cysteine side chain is shown as sticks with atomic coloring: carbon: magenta, sulfur: yellow). Cys-54 and Asp-69 can not form a hydrogen bond as the distance of the sulfur from the oxygen atoms is 5.2 Å and 5.9 Å. (**D**) Scheme of the thiol quantitation assay. Solvent accessible, free cysteines react with the thiol assay reagent and a fluorophore is formed. The observed fluorescent signal is proportional to the amount of free cysteines in the sample. (**E**) Results of thiol quantitation assay of hDUT^F158^^W^ and the hDUT^F158W,Y54C^. (**F**) SDS-PAGE gel of the hDUT^F158W,Y54C^ protein in the absence and presence of 10 mM β-ME. (Full-length gel image is included in a Supplementary Information).

To investigate the effect of Y54C mutation in vitro, we introduced this substitution in the hDUT^F158^^W^ protein. Based on the crystal structures of the human dUTPase Tyr-54 is situated on the surface of the protein (Fig. 3A–C), thus it was straightforward to assume that the mutant cysteine is also solvent accessible. To verify this hypothesis, we performed a thiol quantitation assay (Fig. 3D). Normalized fluorescence of hDUT^F158W,Y54C^ was found to be significantly higher than that of hDUT^F158W^ (cf. Figure 3E), this difference was consistently observable in a wide concentration range. Thus, we concluded that Cys54 is on the surface of the protein.

The reactivity of this residue in the thiol quantitation assay also proposes that probably these cysteines do not participate in extra disulfide bonds. This was confirmed on SDS-PAGE, where we observed similar pattern under non-reducing, non-native conditions as in the case of hDUT^F158^^W^ (Fig. 3F to be compared with Fig. 2D and its analysis).

**Figure 4:**
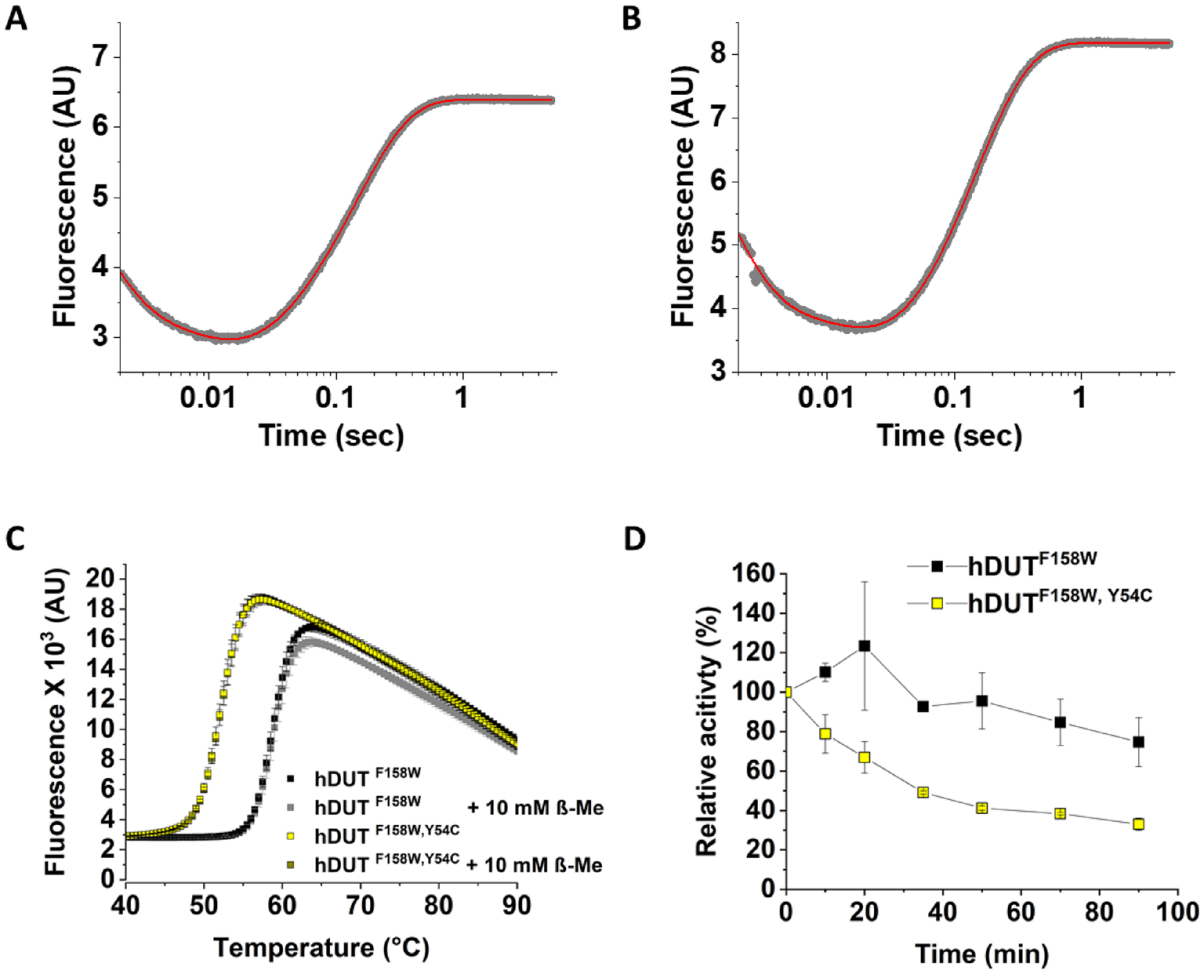
(**A–B**) Representative single turnover fluorescence reaction curves of hDUT^F158^^W,Y54C^ in the presence (**A**) and in the absence (**B**) of 10 mM β-ME. The reaction curves were fitted with triple exponential function (Eq. 1) which yielded the following parameters A_1_ = 3.20 ± 0.14, A_2_ = 1.2 ± 0.01, A_3_ = -4.02 ± 0.003, k_1_ = 877.8 ± 23.9, k_2_ = 118.8 ± 1.4, k_3_ = 7.0 ± 0.01, y_0_ = 6.40 ± 0.0006 for Panel A and A_1_ = 4.61 ± 0.12, A_2_ = 1.74 ± 0.01, A_3_ = − 5.70 ± 0.52, k_1_ = 728.9 ± 13.1, k_2_ = 61.4 ± 0.01, k_3_ = 6.9 ± 0.01, y_0_ = 8.18 ± 0.001 for Panel B (the errors represent the error of the fitted parameters). Several (3–5) single turnover reaction curves were recorded, and the average and standard deviation of the resulted parameters are shown in Table 1. (**C**) Differential Scanning Fluorimetry measurements of the hDUT^F158W^ and hDUT^F158W,Y54C^ proteins in the presence and absence of 10 mM β-ME. Melting temperatures are given in Table 1. (**D**) Stability measurement of the hDUT^F158W^ and hDUT^F158W,Y54C^ proteins. Results of steady-state initial velocity measurements with fixed concentration of dUTP after incubation at 37 °C for various time intervals.

We also performed active site titration and transient kinetic analysis on the mutant enzyme under reducing and non-reducing conditions (Fig. 4A,B, Table 1). The determined k_cat_ value was in good agreement with both the previously published k_cat_ of the hDUT^F158^^W^ enzyme (6.8 ± 2.0 s^−1^) determined from transient kinetic measurements^44^, and with our measurements with the hDUT^F158W^ variant. There was no difference between the reducing and the non-reducing conditions, and again 100% of the protein preparation was active under both conditions.

The observed rate constants of substrate binding were also comparable under the two conditions and they did not differ from the observed rate constants determined for hDUT^F158^^W^ (Table 1).

As previously the mutant enzyme was not characterized, we also performed steady-state activity measurements to determine the Michaelis–Menten parameter of the mutant (Supplementary Fig. 2, Table 1). The determined k_cat_ (6.7 ± 0.7 s^−1^) and K_M_ (2.31 ± 0.98 µM) values did not differ from that of the wild-type and the hDUT^F158^^W^ enzyme^44^. We concluded that the Y54C mutation does not alter the activity and substrate binding properties of the human dUTPase.

To further test the properties of the mutant enzyme we performed differential scanning fluorimetry (DSF) measurements which is applicable for the determination of the melting temperature (T_M_) of the protein referring to its stability. We have found (Fig. 4C) that the mutant enzyme has about 6 °C lower T_M_ (52.5 ± 0.5 °C) than the hDUT^F158^^W^ (58.5 ± 0.5 °C). Addition of β-ME did not influence the thermal unfolding of either protein (Fig. 4C). As T_M_ = 52.5 °C is still relatively high compared to the physiological temperature where the enzyme has to function, we performed stability measurements at 37 °C. For this purpose, we incubated the mutant and the hDUT^F158W^ enzyme at 37 °C for different time intervals and then measured activity (Fig. 4D). We found that the mutant enzyme lost its activity faster than hDUT^F158W^. After 90 min incubation the hDUT^F158W^ variant preserved 75% while the hDUT^F158W,Y54C^ variant only 33% of its activity.

As a structural background, based on the analysis of the interaction network around residue 54 of the wild-type and the Y54C mutant proteins we found only one major change as a consequence of the mutation^61^. In case of the wild-type protein Tyr-54 forms a hydrogen bond with Asp-69, while the cysteine residue cannot establish the same interaction (Fig. 3A–C). Lack of this hydrogen bond in hDUT^F158^^W,Y54C^ may be involved in the observed decrease of thermal stability of the mutant protein.

We concluded based on our in vitro investigation that the hDUT^F158^^W,Y54C^ protein is less stable than the wild-type dUTPase at physiological temperature. This may result in shorter half-life and/or lower activity in vivo.

## Discussion

In the present work we focused on the question whether cysteine oxidation may have any effect on human dUTPase structure and activity. Using a combination of independent methods of structural biochemistry and enzyme kinetics, we report that both under reducing and non-reducing conditions the homotrimeric organization is readily visualized and the enzyme activity is fully preserved.

We observed no significant difference of the catalytic rate constant of the quasi-wild-type human dUTPase, hDUT^F158^^W^ in the presence and absence of 250-times excess reducing agent (ie. 40 μM dUTPase and 10 mM β-ME) (cf. Table 1). In an earlier study steady state activity of a human dUTPase construct (M24-N164) lacking Cys3 was reported to be 8 ± 3 s^-1^ ^16^, which is in good agreement of the data obtained for the hDUT^F158W^ enzyme with transient kinetics experiments (k_cat_ = 6.8 ± 2.0 s^−1^)^44^.

Thus, based on these results we can state that the enzymatic activity of human dUTPase is not dependent on the oxidative state of cysteines. Consequently, regulation of dUTPase activity through redox potential in vivo is unlikely. We therefore respectfully disagree with the previous conclusions of Rotoli et al., who observed marked decrease of enzymatic activity upon addition of 2% β-ME (ca. 289 mM) to the assay buffer. We propose that the observed change is likely to be an artefact caused by the rather high concentration of reducing agent. In the same work Rotoli et al. also observed reduced enzymatic activity in the C3A single point mutant human dUTPase protein, which based on our current results, may have no relation to the redox nature of the protein.

With respect to the proposed presence of dimeric forms of human dUTPase, we note that the method of choice in native mass spectrometry is the Electrospray Ionization (ESI)-MS for detection of protein oligomers^62^. In the present study we have used ESI–MS and found that the majority of the protein sample corresponds to the trimeric assembly, whereas in the previous report MALDI-TOF was used^41^, that is much less applicable for studying native protein complexes.

Here we also report, that the intriguing Y54C mutation identified in patients of a monogenic syndrome associated with diabetes and bone marrow failure, also did not change the enzyme activity at room temperature but led to decreased thermal stability. There might be two different explanations for the observed pathological effects in patients carrying this mutation. On the one hand the decreased thermal stability may result in shorter half-life, and in insufficient in vivo activity. In some other systems as well, different enzyme mutations leading to impaired cellular functions may similarly involve temperature sensitivity^63,64^.

On the other hand, the mutation may perturb protein–protein interactions. This hypothesis is based on the premise that Cys54 is located on the surface of the protein. The significantly increased amount of reactive cysteines observed in the thiol reactivity assay of hDUT^F158^^W,Y54C^ compared to that of hDUT^F158W^ indicate that the mutant cysteine is indeed on the surface of the protein, thus could potentially interfere with binding of the human dUTPase to other proteins in vivo*.*

Finally based on our results we conclude that cysteines do not act as redox switches in the human dUTPase. Introduction of cysteine mutation at position 54 of the nuclear isoform of the human dUTPase alters the stability of the protein, which could be the reason why this mutation leads to a monogenic syndrome associated with diabetes and bone marrow failure.

## Methods

### Cloning and mutagenesis

Site-directed mutagenesis was performed by the QuikChange method (Stratagene) and was verified by sequencing. The enzyme conferring a tryptophan sensor in the active site (hDUT^F158^^W^) was used as wild-type^16,43,44,60,65,66^. The Tyr54 to Cys mutant was created within this construct using the following forward (FW) and reverse (REV) primers: Y54C_FW: 5ʹ CGACCTGTACAGTGCCT**G**TGATTACACAATACCACCTATGG 3ʹ and Y54C_REV: 5ʹ GCTGGACATGTCACGGTCTCTAATGTGTTATGGTGGATACC 3ʹ. Thus the hDUT^F158W^ and the hDUT^F158W,Y54C^ constructs have the same sequence as the nuclear isoform of the human dUTPase (Fig. 1A, nDut) except the indicated mutations and an N-terminal polyhistidine tag (MAHHHHHHVGT).

### Protein expression and purification

Expression and purification of the human dUTPase proteins were done as described previously in ^66^. Briefly, the hDUT^F158^^W^ and the hDUT^F158W,Y54C^ enzymes were expressed in BL21 Rosetta (pLysS) cells (Novagen). The protein expression was induced by the addition of 500 µM isopropyl β-D-1-thiogalactopyranoside at OD = 0.5 and was conducted for 4 h at 37 °C. The proteins were purified on His-NTA column as described in ^66^, except that before the elution (500 mM imidazole) a two-step imidazole washing was done, by 75 mM and 100 mM imidazole. The proteins were purified either in the presence or in the absence of reducing agent. As a reducing agent 10 mM 2–mercaptoethanol (β–ME) was used. The protein concentration was measured by UV absorbance. Extinction coefficients were calculated based on the amino acid sequence using the ProtParam tool (http://web.expasy.org/protparam/). Extinction coefficients for the proteins were: λ_280_ = 15,930 M^−1^ cm^−1^ and λ_280_ = 16,055 M^−1^ cm^−1^ for the hDUT^F158W^ (reduced/non-reduced), λ_280_ = 14,440 M^−1^∙cm^−1^ and λ_280_ = 14,690 M^−1^∙cm^−1^ for the hDUT^F158W,Y54C^ (reduced/non-reduced). Protein concentration is given in monomer/subunit concentration in every case. All measurements were carried out in a buffer comprising 20 mM HEPES pH 7.5, 100 mM NaCl, 2 mM MgCl_2_ and 10 mM β-ME (“assay buffer”) if not stated otherwise.

### Enzyme activity assay

#### Michaelis–Menten enzyme kinetics

Proton release during the transformation of dUTP into dUMP and PPi was followed continuously at 559 nm at 20 °C using a JASCO-V550 spectrophotometer^67,68^. Reaction mixtures contained 10 nM enzyme and varying concentrations of dUTP. The reaction was started with the addition of dUTP. Initial velocity was determined from the slope of the first 10% of the progress curve. Initial velocities were plotted against substrate concentration and the results were fitted with the Michaelis–Menten equation.

#### Investigation of enzyme stability by activity measurement

This measurement was done with the same assay, except that fixed concentration of dUTP (30 µM) was used, and the enzyme was pre-incubated at 37 °C for different time intervals (0–90 min).

### Transient kinetics experiments

Fluorescence stopped-flow measurements were carried out using an SX20 (Applied Photophysics, UK) stopped-flow instrument, following tryptophan fluorescence at 20 °C, as described previously^44,60,65,66^. Equal volumes (50 μl) of 40 μM dUTPase enzyme and 40 μM dUTP solutions (both diluted in assay buffer) were mixed and typically 3–5 traces were collected and averaged. A triple exponential function (Eq. 1) was fitted to the averaged traces to determine the catalytic constants, and the observed binding rates at the given substrate concentration based on Tóth et al. ^44^.

1 $$F = A_{1} *e^{{ - k_{1obs} x}} + A_{2} *e^{{ - k_{2obs} x}} + A_{3} *e^{{ - k_{3obs} x}} + y0,$$

where F is the observed fluorescence, x is the variable (time), A_1,2,3_ are the amplitudes, k_1,2,3obs_ are the rate constants of the observable fluorescence phases, while y_0_ is the y offset.

### Differential Scanning Fluorimetry (DSF)

DSF assays were carried out on a Bio-Rad CFX96 qPCR in FrameStar® 96 Well Skirted PCR Plates with black wire and white wells sealed with Eppendorf adhesive PCR films. Reactions were performed in a total volume of 25 μl containing 500 × diluted Sypro® Orange dye. Samples were heated from 25 to 90 °C. The speed of heating was 2 °C/minute. The protein concentration was 0.8 mg/ml in the measurements. The melting point was determined by taking the negative derivate of the curves. β-ME was added as indicated on Fig. 4C.

### Thiol quantitation assay

Reactive thiols were measured by MAK151 Sigma Fluorometric Thiol Quantitation Kit according to manufacturer instructions. Briefly 10 μl of the protein samples was added to 10 μl of thiol reagent in a well of a low volume black bottom plate (CLS4514 Sigma). Samples were mixed and then kept in dark for 30 min. Fluorescence intensity was measured at λ_ex_ = 490 nm/ λ_em_ = 535 nm by Spectramax M5 Plate reader (Molecular Devices). Fluorescence values were normalized by subtraction of the fluorescence measured for the buffer. Each sample were measured in triplicates.

### Electrospray ionization mass spectrometry

The oligomerization state of the human dUTPase was studied by a Waters QTOF Premier mass spectrometer (Waters, Milford, MA, USA) equipped with electrospray ionization source (Waters, Milford, MA, USA) operated in positive ion mode. Purified hDUT samples of 20 µM were subjected to buffer exchange to 200 mM NH_4_HCO_3_ buffer performed with Vivaspin® 500 Polyethersulfone centrifugal concentrators of 10 kDa weight cutoff. Mass spectra were measured under native conditions: namely, the ions were generated from aqueous 5 mM NH_4_HCO_3_ buffer solution (pH = 7.5) containing hDUT at ca. 0.5 µM monomer concentration, which conditions favor the transfer of the protein complexes from solution into the gas phase. The capillary voltage was 2600–2800 V, the sampling cone voltage was 125 V and the temperature of the source was set at 80 °C, collision cell pressure was 3.43 × 10^–3^ mbar and ion guide gas flow was 35 ml/min. Mass spectra were recorded applying the software MassLynx 4.1 (Waters, Milford, MA, USA) in the 1000–8000 m/z mass range^43^.

## Supplementary Information

Supplementary Information.
