# Author Correction: Redox status of cysteines does not alter functional properties of human dUTPase but the Y54C mutation involved in monogenic diabetes decreases protein stability

**DOI:** 10.1038/s41598-021-01241-2

**Published:** 2021-10-29

**Authors:** Judit Eszter Szabó, Kinga Nyíri, Dániel Andrási, Judit Matejka, Olivér Ozohanics, Beáta Vértessy

**Affiliations:** 1 Institute of Enzymology, RCNS, Eötvös Loránd Research Network, Budapest, Hungary; 2 Department of Applied Biotechnology and Food Sciences, Budapest University of Technology and Economics, Budapest, Hungary; 3 Department of Biochemistry, Institute of Biochemistry and Molecular Biology, Semmelweis University, Budapest, Hungary

Correction to: *Scientific Reports* 10.1038/s41598-021-98790-3, published online 28 September 2021

The original version of this Article contained an error in the spelling of the author Olivér Ozohanics which was incorrectly given as Olivér Ozahonics. The original Article and accompanying Supplementary Information file have been corrected.

