# Supplementary material for: Redox status of cysteines does not alter functional properties of human dUTPase but the Y54C mutation involved in monogenic diabetes decreases protein stability
Source: Sci Rep. 2021 Sep 28;11:19197. doi: 10.1038/s41598-021-98790-3 (PMC8478915; doi:10.1038/s41598-021-98790-3)
Supplement: Supplementary file 1 — Supplementary Information. [file 41598_2021_98790_MOESM1_ESM.docx]

**Supplementary Information**

**Redox status of cysteines does not alter functional properties of human dUTPase but the Y54C mutation involved in monogenic diabetes decreases protein stability**

Judit Eszter Szabó^1,2,^*^#^, Kinga Nyíri^1,2#^, Dániel Andrási^2^, Judit Matejka^1,2,^, Olivér Ozohanics^3^, Beáta Vértessy^1,2^*

^1^Institute of Enzymology, RCNS, Hungarian Academy of Sciences, Budapest, Hungary;

^2^Department of Applied Biotechnology and Food Sciences, Budapest University of Technology and Economics, Budapest, Hungary

^3^Department of Biochemistry, Institute of Biochemistry and Molecular Biology, Semmelweis University, Budapest, Hungary

**# equal contribution**

*****Correspondence: vertessy@kutatok.org; szabo.judit.eszter@ttk.hu


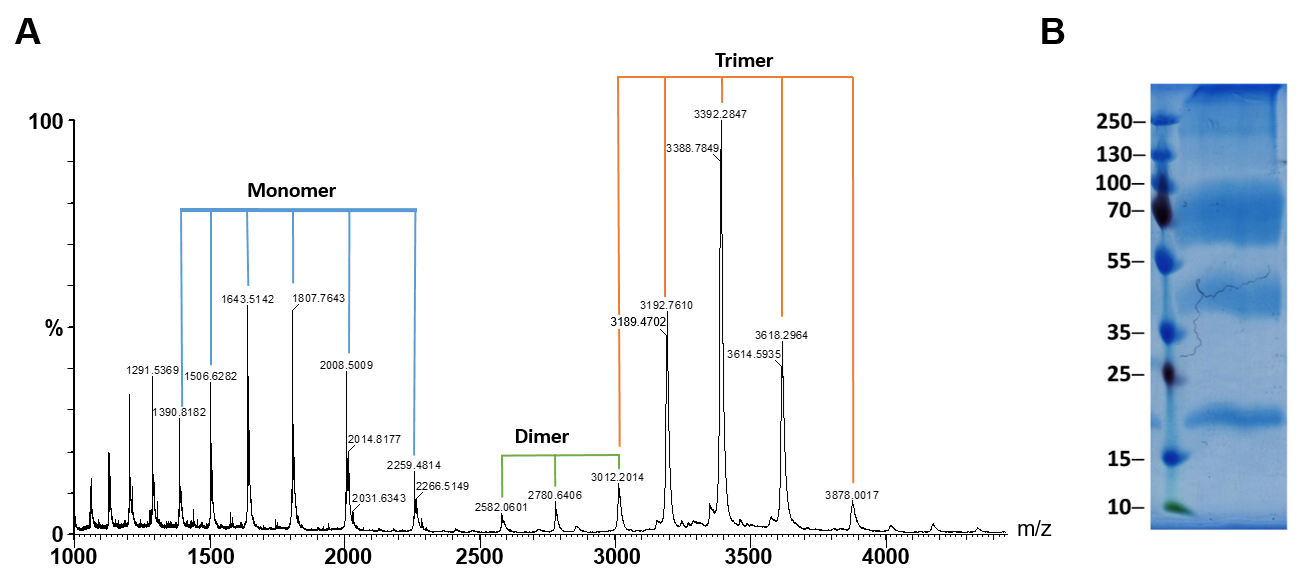


**Fig S1. A)** Annotated mass spectra of the human dUTPase protein obtained under native electrospray conditions. Peaks corresponding to monomer, dimer and trimer species are framed. Based on the integrated area of the peaks abundance of the monomer, dimer and trimer forms are 36 %, 3%, 61% respectively, with ±1% accuracy. **B) SDS-PAGE analysis of recombinantly expressed and purified human dUTPase (hDUT- nuclear isoform) crosslinked with disuccinimidyl suberate.** Trimer, dimer and monomer forms are prevalent, while some aggregate has also been formed. The observed broadening of the bands is due to perturbed relaxation of the crosslinked species by covalent-crosslinks. Full-length gel image is included at the end of this document.


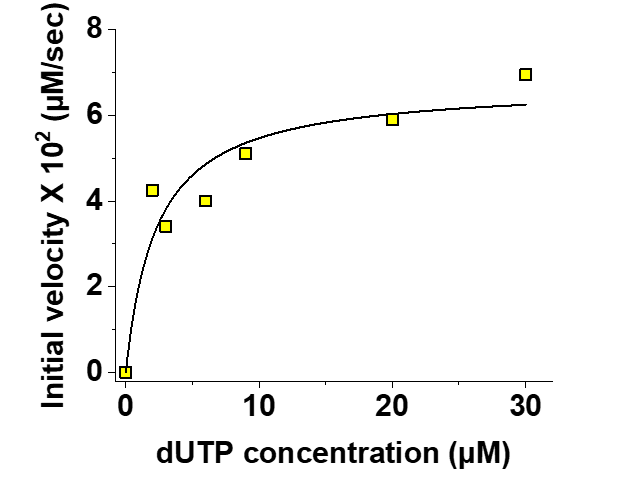


**Fig S2.** Steady-state initial velocity measurement of the hDUT^F158W,Y54C^ protein with various concentration of dUTP substrate. The fitting of the Michaelis-Menten equation yielded the following parameters: V_max_=0.067 ± 0.007 µM/s, K_M_=2.31 ± 0.98 µM. The calculated k_cat_ = 6.7 ± 0.7 s^-1^

## Chemical crosslinking

Crosslinking experiments were performed as described earlier^61^. Human dUTPase of 80 μM concentration was incubated with 20 mM disuccinimidyl suberate (DSS) at 20 °C for one hour. The crosslinking reactions were quenched with the addition of 5 μl 100 mM (pH=7.5) TRIS buffer to 40 μl of samples and then those were analyzed by SDS-PAGE after electrophoresis for 45 min at 200 V using a two‑phase polyacrylamide gel; in which acrylamide concentration was 4% in the stacking gel and 12% in the resolving gel. Page Ruler prestained protein ladder (Thermo Fisher) was used as a molecular weight marker. Page Blue protein staining solution containing Coomassie Brilliant Blue dye was used to stain the gel.

**Full length gel pictures of Figure 2D and Figure 3F of the main text and Figure S1B.**

**
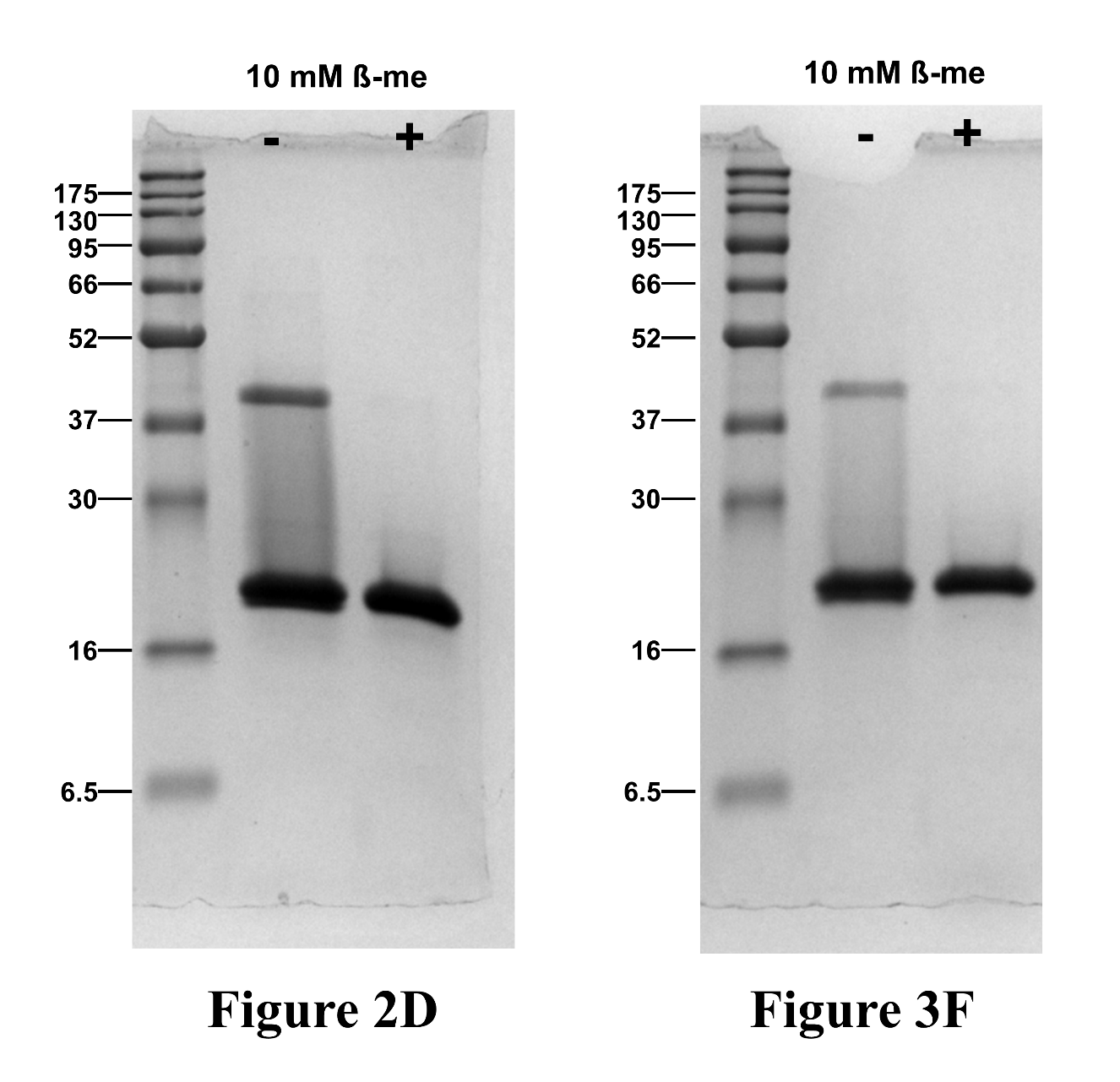
**

**
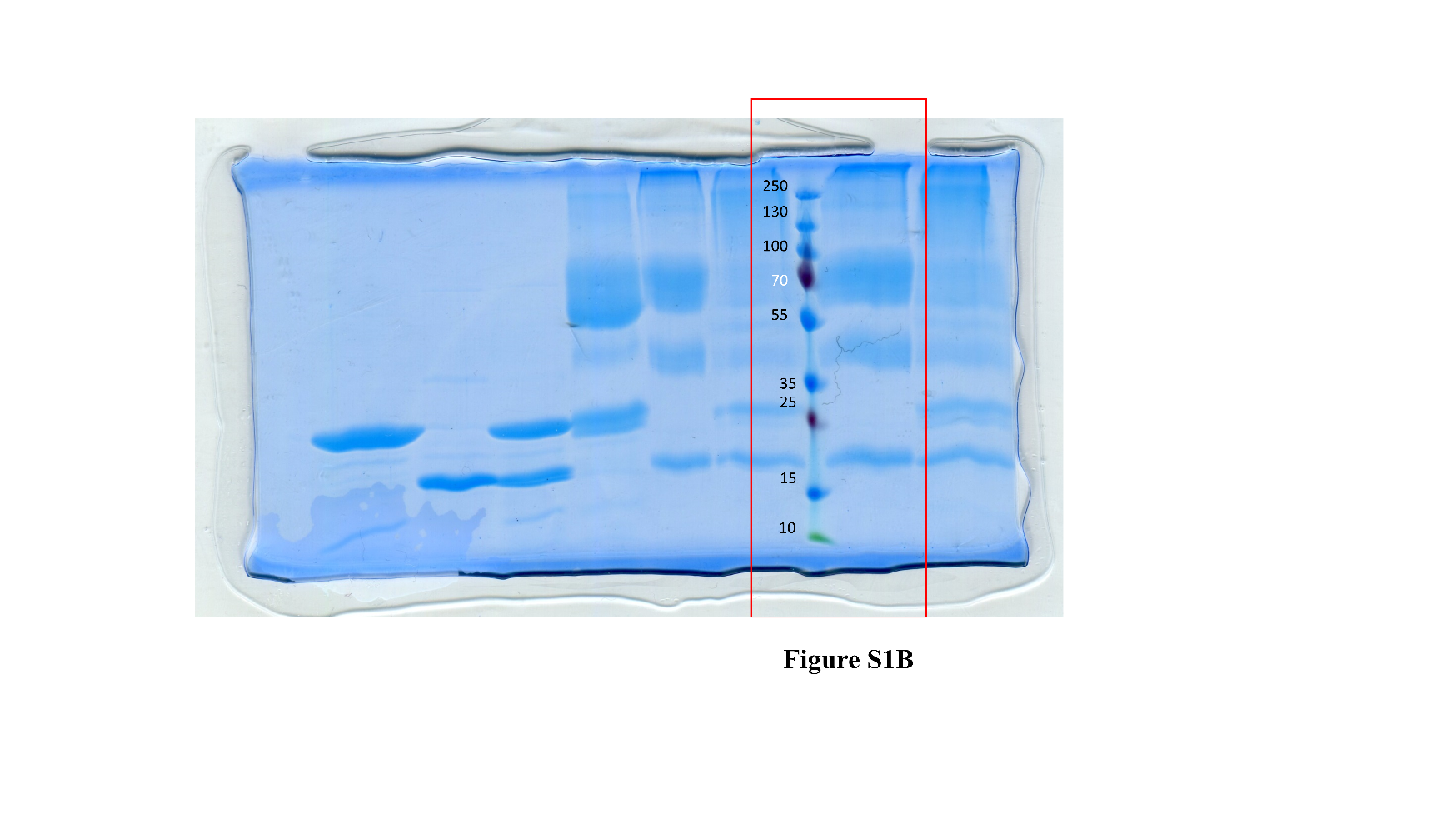
**
